# Topical diclofenac gel for actinic damage in patients with alopecia

**DOI:** 10.1016/j.jdcr.2025.11.017

**Published:** 2025-11-20

**Authors:** Milan M. Hirpara, Colin Kincaid, Rachel Greene, Sarah Choe, Christine T. Pham, Natasha A. Mesinkovska

**Affiliations:** a Department of Dermatology, University of California, Irvine, Irvine, California; b California University of Science and Medicine, School of Medicine, Colton, California

**Keywords:** actinic damage, actinic keratosis, alopecia, androgenetic alopecia, diclofenac, field therapy, inflammation, lichen planopilaris

## Introduction

Chronic inflammation is a central driver of hair loss in several alopecia subtypes. In scarring alopecias such as lichen planopilaris (LPP), inflammation results in irreversible follicle destruction, whereas in androgenetic alopecia (AGA), perifollicular microinflammation contributes to progressive miniaturization.^1^^,^^2^ Previous literature has suggested that patients with scarring alopecias are at an increased risk for the development of squamous cell and basal cell carcinomas due to this prolonged inflammation to exposed areas of the scalp.^1^

To mitigate this carcinogen risk, various field therapy options are available, including 5-fluorouracil (5-FU), photodynamic therapy (PDT), and diclofenac. Selection of field treatment can be challenging on scalp hair bearing areas. 5-FU’s mechanism as an antimetabolite raises concerns and has been reported to induce hair loss.^3^ PDT has been evaluated for its effects on hair regrowth with mixed results in AGA and alopecia areata (AA).^4^ Paradoxically, PDT has been shown effective in hair removal in patients with hirsutism and hypertrichosis.^5^

Diclofenac, a nonsteroidal anti-inflammatory drug, has been shown to reduce inflammation and hair loss while treating actinic damage.^6^, ^7^, ^8^ Herein, we present a case series of 5 patients with alopecia and concomitant actinic scalp damage treated with topical diclofenac 1% to 3% gel (Table I). Patients showed clinical improvement in actinic damage, and in several cases, evidence of hair regrowth was observed in the treated areas. We also broaden this investigation with a review of the literature on the efficacy of diclofenac in regrowing hair in patients with alopecia (Table II).

**Table I:** Clinical characteristics, treatment regimens, and outcomes in patients with alopecia and actinic damage treated with topical diclofenac gel

Patient	Age (y)	Sex	Dx	Therapy regimen	Additional AK treatments	Hair loss treatment	Redness and scale change∗	Hair growth change∗
1	78	F	LPP	Topical diclofenac 1% gel, TID (3 mo)	- Oral niacinamide 500 mg BID - Liquid nitrogen	- Oral minoxidil 1.25 mg QD - ILK (2.5 and 5 mg/mL)	+2	+2
2	45	M	AGA	Topical diclofenac 3% gel, TIW (3 mo)	None	- Oral finasteride 5 mg QD	+2	+1
3	69	F	AGA	Topical diclofenac 1% gel, TID (3 mo)	Liquid nitrogen	- Oral finasteride 2.5 mg QD - Oral minoxidil 2.5 mg QD	+3	+2
4	73	F	AGA	Topical diclofenac 1% gel, BIW (4 mo)	Liquid nitrogen	- Oral minoxidil 0.625 mg QD - Oral spironolactone 100 mg QD	+2	+1
5	72	F	LPP	Topical diclofenac 3% gel, QD (6 mo)	None	- Oral minoxidil 1.25 mg QD - ILK (2.5 and 5 mg/mL)	+2	+1

*AGA*, Androgenetic alopecia; *AK*, actinic keratosis; *BID*, 2 times a day; *BIW*, 2 times weekly; *Dx*, diagnosis; *F*, female; *ILK*, intralesional triamcinolone; *LPP*, lichen planopilaris; *M*, male; *QD*, daily; *TID*, 3 times daily; *TIW*, 3 times weekly.

∗ Redness and scale change and hair growth change were evaluated by the single treating dermatologist. The evaluation occurred at the end of the prescribed treatment regimen and was compared with baseline using standardized clinical photographs. Change was recorded on a 7-point Clinician Assessment Scale: −3 = Significant Worsening, −2 = Moderate Worsening, −1 = Slight Worsening, 0 = Same, +1 = Slight Improvement, +2 = Moderate Improvement, +3 = Significant Improvement.

**Table II:** Published reports on the efficacy of diclofenac in alopecia

Study	Patients, *n* (M, F)	Age, mean ± SD (y)	Dx	Treatment regimen	Concurrent hair loss regimen	Clinical course
Collgros (2015)^6^	3 (3 M, 0 F)	76.3 ± 2.5	AGA	Topical 3% diclofenac gel, QD (4-6 mo)	None	- 6 mo: Evidence of terminal hair growth over nonprior haired biparietal area - Patient 1 followed for 4 y because of his actinic keratoses and showed continuous hair growth
Sharquie (2006)^7^	20∗	19.7 ± 11.5	AA	Topical 1% diclofenac gel, BID (2 mo)	None	- Of 32 AA patches identified, 21 (66%) patches showed complete hair regrowth and 3 (9%) patches revealed partial response by the second month. - 22 (69%) patches showed complete regrowth by 4 mo. - 9 (28%) patches showed no response at 4 mo.
Seifian (2024)^8^	11 (11 M)	--†	AGA	Topical 3% diclofenac gel, BID (4 mo)	None	- No significant difference in the number of hair follicles in a 2.2-cm region within the frontal and vertex areas between baseline and final follow-up visits.

*AA*, Alopecia areata; *AGA*, androgenetic alopecia; *BID*, 2 times a day; *Dx*, diagnosis; *F*, female; *M*, male; *QD*, daily; *SD*, standard deviation.

∗ Sex of patients was not specified.

† Average age of patients within this cohort was unspecified.

## Case series

### Case 1

**Fig 1:**
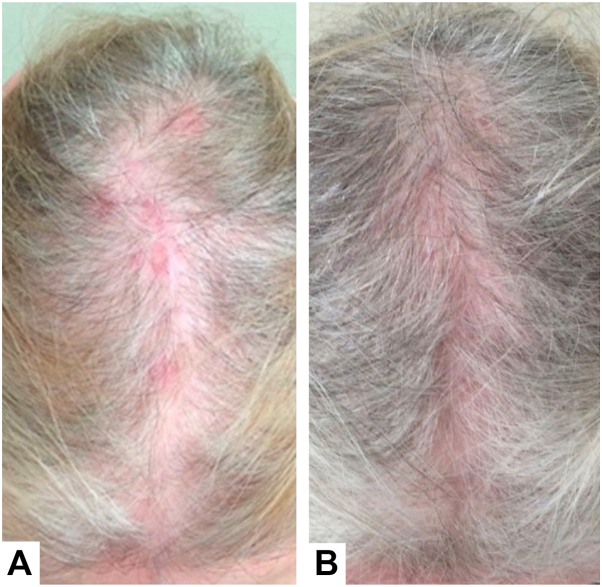
**A,** Diffuse scalp erythema involving the frontal and vertex scalp (78-year-old female, lichen planopilaris). **B,** Improvement of scalp erythema and vertex hair density following 3 months of topical diclofenac 1% gel applied 3 times daily.

**Fig 2:**
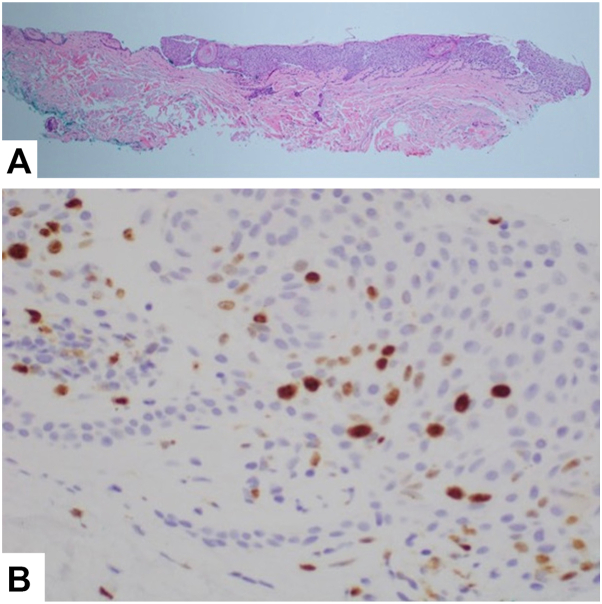
Histopathological findings of the right vertex scalp for patient 1. **A,** H&E stain of scalp punch biopsy showing atypical keratinocytes throughout the entire thickness of the epidermis, 4×. **B,** Ki67 stain shows an increased proliferation index in the upper layers of the epidermis consistent with an actinic keratosis/early squamous cell carcinoma in situ, 40×. *H&E*, Hematoxylin and eosin.

A 78-year-old female with biopsy-proven LPP presented to the clinic due to the lack of improvement in her hair loss despite treatment with oral hydroxychloroquine 200 mg twice daily, oral doxycycline 100 mg twice daily, and intralesional triamcinolone (2.5 and 5 mg/mL) every 3 months. On physical examination, there was widespread erythema and decreased scalp hair density most prominent on the vertex scalp with sparse lateral eyebrows (Fig 1, *A*). Dermatoscopic examination revealed significant folliculitis and perifollicular scale. A punch biopsy of the right vertex scalp revealed decreased hair follicles. Elastin stain failed to demonstrate a scarring process and there was no evidence of an active inflammatory process. Atypical keratinocytes were incidentally identified throughout the epidermis and a Ki67 staining showed an increased proliferation index in the upper layers of the epidermis (Fig 2). These findings were consistent with a diagnosis of hyperplastic actinic keratosis (AK) with the possibility of early squamous cell carcinoma in situ. The patient was diagnosed with quiescent LPP and general scalp inflammation secondary to actinic damage.

The patient was prescribed topical diclofenac 3% gel twice daily on the frontal and vertex scalp for 3 months as field therapy for actinic damage. Due to insurance coverage issues, this therapy was substituted with topical diclofenac 1% gel 3 times daily. Treatment also included oral niacinamide 500 mg twice daily and oral minoxidil 1.25 mg daily with intralesional triamcinolone (2.5 and 5 mg/mL) administered every 3 months as needed for scalp itch and pain. After 3 months, the patient exhibited marked improvement of scalp erythema without any signs of further hair loss. In fact, noticeable hair regrowth was observed in prior areas of actinic damage along the vertex scalp (Fig 1, *B*). Residual scalp AKs were treated with cryotherapy at follow-up visits. Given the hair regrowth, the patient was recommended to continue topical diclofenac 1% gel 3 times weekly to areas of hair loss along with her oral and intralesional therapies. At 2-year follow-up, she demonstrated sustained hair regrowth.

### Case 2

**Fig 3:**
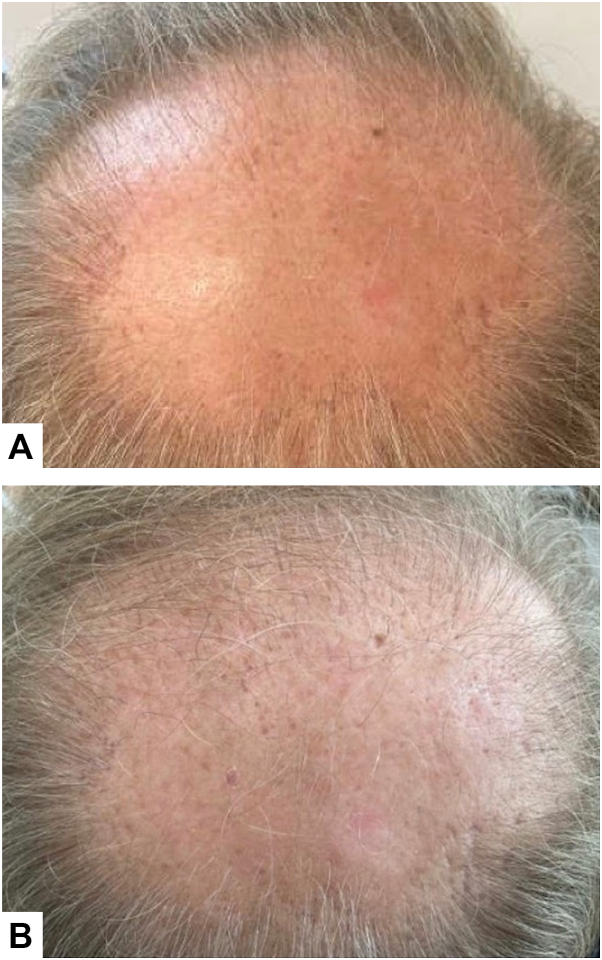
**A,** Diffuse scalp erythema and actinic damage involving the vertex scalp (45-year-old male patient, androgenetic alopecia). **B,** Improvement in actinic damage and increased vellus hair density over the vertex following 3 months of topical diclofenac 3% gel applied 3 times weekly.

A 45-year-old male with a 6-year history of AGA and robotic hair transplant 3.5 years prior presented to the clinic for evaluation of scalp flaking and irritation. Medications included oral finasteride 5 mg daily, continued for the last 6 years. On examination, there was widespread scale and diffuse hair thinning most pronounced over the vertex scalp, with multiple erythematous, scaly papules over the vertex, consistent with AKs (Fig 3, *A*). The patient was prescribed topical diclofenac 3% gel twice daily applied to the vertex scalp for 3 months. After 1 month, the patient exhibited resolution of AKs and regrowth of vellus hairs on the vertex scalp (Fig 3, *B*).

Diclofenac was continued for an additional 2 months, alongside oral finasteride, and the patient endorsed continued hair growth.

### Case 3

A 69-year-old female with biopsy-proven AGA presented for routine hair loss follow-up. The patient’s treatment regimen included oral minoxidil 2.5 mg daily and oral finasteride 2.5 mg daily. On examination, there was diffuse hair thinning over the frontal and vertex scalp with photodamage and overlying erythematous scaly papules noted on the frontal scalp. The patient was prescribed 3% topical diclofenac gel twice daily to areas of actinic damage on the frontal hairline for 3 months while continuing her hair loss regimen unchanged. Following denial by insurance, the patient was switched to 1% topical diclofenac gel 3 times daily for 3 months. At her 3-month follow-up visit, physical examination revealed new hair growth and improvement of actinic damage on the frontal scalp. Residual AKs of the frontal hairline were treated with cryotherapy.

### Case 4

A 73-year-old female with a 3-year history of biopsy-proven AGA presented for routine evaluation and red blotches on the central scalp. Her current medications included oral minoxidil 0.625 mg daily and oral spironolactone 100 mg daily. Physical examination revealed multiple rough, erythematous papules with gritty scale involving the vertex scalp, consistent with AKs. Consequently, she was prescribed 1% topical diclofenac gel twice daily applied to the vertex scalp with no changes to her hair loss regimen. At 4-month follow-up, there was significant reduction in actinic damage over the treated areas with emerging short hairs along the vertex and midline part. Remaining AKs were treated with cryotherapy at the same visit.

### Case 5

A 72-year-old female with a history of LPP presented for routine follow-up for chronic hair loss. Her treatment regimen included oral minoxidil 1.25 mg daily and intralesional triamcinolone (2.5 and 5 mg/mL) every 3 months as needed. On examination, she demonstrated diffuse hair thinning involving the frontal, vertex, and occipital scalp with erythematous, scaly papules noted over the vertex, clinically suggestive of AKs. She was started on 3% topical diclofenac gel applied once daily to the vertex scalp while maintaining her existing hair loss regimen. At her 6-month follow-up, the AKs had resolved, and early signs of hair regrowth were observed in the treated areas.

## Discussion

Treatment of scalp AKs in patients with coexisting alopecia presents a unique therapeutic challenge. In our alopecic patients, the choice of a field therapy had to account for the potential to worsen hair loss compared to traditional field therapy. Both 5-FU and PDT pose a risk. In mice models, 5-FU was found to induce anagen-to-catagen transition and delayed anagen initiation by inhibiting angiogenesis and inhibiting beta-catenin signaling resulting in cell cycle arrest.^9^ Although PDT has been suggested to possibly stimulate hair regrowth, studies in patients with AGA and AA have not shown significant hair growth following treatment.^4^^,^^10^ Contrarily, some studies have suggested PDT may induce hair loss at treatment sites.^5^ In patients with hypertrichosis or hirsutism, PDT treatment showed a 75% or more hair reduction at 12 months in 43% of treated sites.^11^ Authors suggested that PDT induced a cytotoxic effect involving interleukin-1 and tumor necrosis factor-α at the hair follicle bulge and dermal papilla, inhibiting hair growth.^11^

Previous literature has reported promising results on the use of topical diclofenac in stimulating hair regrowth. Collgros et al described 3 elderly men with AGA who experienced sustained terminal hair regrowth after applying topical diclofenac 3% gel daily for 4 to 6 months.^6^ Similarly, Sharquie et al reported complete regrowth in 66% of AA patches treated with topical diclofenac 1% gel twice daily for 2 months.^7^ This growth was sustained even 2 months after completing the diclofenac course with only 28% of patches showing no growth at 4 months. Importantly, the patients from these 2 studies were not receiving any concurrent treatments. However, 1 prospective study in 11 men with AGA found no significant change in follicular density after 4 months of topical diclofenac 3% gel, indicating that treatment response may vary depending on individual factors such as alopecia subtype, severity, and the presence of underlying scalp inflammation (Table II).^8^

The therapeutic effects of diclofenac in both AKs and alopecia may be attributed to its dual anti-inflammatory mechanism. Diclofenac disrupts the arachidonic acid pathway, a major inflammatory pathway, through inhibition of the cyclooxygenase-2 (COX-2) enzyme. This inhibition causes a decrease in the production of various prostaglandins which are synthesized from the conversion of arachidonic acid by COX-2. Both COX-2 and prostaglandins have been suggested as key regulators of tumorigenesis and by deregulating this pathway, diclofenac can decrease the progression of AKs to squamous cell carcinomas.^12^ Prostaglandins play a role in hair growth and significantly upregulated prostaglandin D2 levels are found in the scalp skin biopsies of patients with AGA.^13^^,^^14^ Given the anti-inflammatory benefits, diclofenac gel was selected as the treatment for our patients’ scalp actinic damage and concomitant hair loss.

Our case series highlights the utility of topical diclofenac 1% to 3% gel as a safe field therapy for AKs in hair-bearing areas, where treatment options are often limited by the risk of exacerbating hair loss. Although all patients experienced hair growth in treated areas, concurrent alopecia therapies make it challenging to isolate the effects of diclofenac on hair regrowth. However, no patient experienced increased shedding or expansion of alopecic areas while using diclofenac. No adverse events were observed, including erosive pustular dermatosis, a rare cutaneous complication, more frequently seen on chronically photodamaged scalps and may be triggered by topical chemotherapeutic agents.

Other limitations include heterogeneity in patient alopecia subtypes, including AGA and LPP, which may have differing baseline inflammation and treatment responsiveness. Diclofenac formulations and dosing also varied due to insurance constraints, ranging from a concentration of 1% or 3% gel at different frequencies. Larger controlled studies are needed to standardize endpoints and evaluate the independent effect of diclofenac on hair loss.

Diclofenac demonstrated efficacy in reducing actinic damage without worsening alopecia. These findings underscore the importance of tailoring field therapy to patient-specific and disease-specific factors. Clinicians should consider diclofenac gel in patients with concomitant scalp AKs and alopecia.

## Conflicts of interest

None disclosed.
